# Survival tactics of *Bacillus licheniformis* KNP under hexavalent chromium stress: a study of detoxification and chemotactic responses

**DOI:** 10.3389/fmicb.2025.1646518

**Published:** 2025-09-05

**Authors:** Akansha Garg, Mushtaq Ali, Aiman Ahmad, Prerna Chauhan, Ashish Kumar, Rupali Mishra, Vimal Kumar Dubey, Alok Srivastava, Sanjay Kumar Garg, Pankaj Kumar Arora

**Affiliations:** ^1^Department of Plant Science, MJP Rohilkhand University, Bareilly, India; ^2^Department of Microbiology, MJP Rohilkhand University, Bareilly, India; ^3^Department of Environmental Microbiology, Babasaheb Bhimrao Ambedkar University, Lucknow, India; ^4^Faculty of Agriculture, Mahayogi Gorakhnath University, Gorakhpur, India

**Keywords:** chemotaxis, detoxification, chromium, heavy metal, *Bacillus*

## Abstract

Hexavalent chromium [Cr(VI)] pollution is a serious environmental issue because it is highly toxic and persistent. We aimed to investigate the growth, Cr(VI) reducing capacity, and chemotaxis of *Bacillus licheniformis* strain KNP under the stress of Cr(VI) in the form of K_2_Cr_2_O_7_ (250–1,000 ppm). Bacterial growth decreased as Cr(VI) concentration increased, with a maximum at 250 ppm K_2_Cr_2_O_7_ and strong inhibition at 1,000 ppm K_2_Cr_2_O_7_. Significantly, strain KNP completely reduced Cr(VI) in the form of 500 ppm K_2_Cr_2_O_7_ within 48 h. Fourier Transform Infrared spectroscopy (FTIR) showed biochemical changes of functional groups present on bacterial cell wall due to interaction with chromium. A scanning electron microscope (SEM) and energy dispersive X-ray spectroscopy (EDX) analysis confirmed the accumulation of Cr on the surface of bacteria with morphological changes. Strain KNP also showed negative chemotaxis away from Cr(VI) and positive chemotaxis toward glucose, as indicated by drop plate and chemical plug assays. Genomic analysis revealed major chemotaxis-related genes that play a role in Cr(VI) sensing and avoidance, indicating a complex survival strategy. These results indicated that *B. licheniformis* strain KNP is a good candidate for bioremediation purposes, providing an effective combination of Cr(VI) detoxification and tactical bacterial migration in polluted environments.

## Introduction

Chromium (Cr) is a widespread heavy metal in the environment, which primarily occurs in two oxidation states: trivalent chromium [Cr(III)] and hexavalent chromium [Cr(VI)] (Abbas et al., 2025; Kumari et al., 2025). Though Cr(III) is an essential trace element with negligible toxicity, Cr(VI) is very toxic, carcinogenic, and causes significant environmental and health risks due to its high solubility and mobility in aquatic systems (Xing et al., 2025). Industrial operations like stainless steel production, leather tanning, and mining are major contributors to Cr(VI) contamination, which underscores the necessity for efficient remediation strategies (Xiang et al., 2025; Xing et al., 2025).

The biotransformation of chromium is a crucial microbially mediated process wherein Cr(VI) is detoxified through reduction to the less toxic and insoluble Cr(III), which subsequently precipitates and becomes less bioavailable (Xing et al., 2025). Various bacterial, fungal, and archaeal strains have evolved enzymatic mechanisms to counteract Cr(VI) toxicity via intracellular and extracellular reduction mechanisms. These detoxification pathways generally include chromate reductases, electron transport chains, and oxidative stress response systems, allowing microorganisms to tolerate and metabolize Cr(VI) in a wide variety of environmental settings (Xing et al., 2025). A comprehensive understanding of these microbial metabolic pathways is fundamental to optimizing bioremediation strategies for chromium-contaminated environments (Saxena et al., 2025).

The comparative analysis of bacterial species involved in Cr(VI) reduction reveals a diverse array of efficiencies across genera. *Bacillus licheniformis* stands out for its broad reduction range, effectively reducing 20–1,500 ppm Cr(VI) within 24–72 h (Kavitha et al., 2011), whereas its thermotolerant strain B22 tolerates up to 100 ppm (Doganli and Dogan, 2014). Other *Bacillus* species also demonstrate significant reduction capabilities: *B. cohnii* SR2 and *B. licheniformis* SR3 achieved 94%−95% reduction of 100 ppm Cr(VI) within 25 h (Sarankumar et al., 2020). *B. megaterium, B. carbonip*hilus, *B. subtilis*, and *B. licheniformis* showed tolerance and reduction across 125–500 ppm concentrations (Pinki et al., 2021). *B. wiedmannii* S1 showed a 70% reduction of Cr(VI) when the initial concentration was 200 μg/ml (Adhikary et al., 2025). *Bacillus tropicus* CRB14 reduced 86.57% Cr(VI) within 96 h at higher concentrations (Tuli et al., 2024). *B. paramycoides* S48 achieved a 90% reduction of chromium within 48 h at concentrations ranging from 25 to 50 mg/L. However, the reduction efficiency progressively declined as the concentration of K_2_Cr_2_O_7_ increased from 100 to 500 mg/L (Kalsoom et al., 2025). Notably, *B. vallismortis* and *B. haynesii* formed biofilms in response to chromium stress, which contributed 60%−99% Cr(VI) reduction (Maurya et al., 2022), suggesting biofilm-mediated detoxification as a potent strategy.

In contrast, *Cellulosimicrobium* species exhibit high tolerance and reduction efficiency, with SCRB10 tolerating up to 800 ppm and reducing 96.98% of 100 ppm Cr(VI) in 96 h (Bharagava and Mishra, 2018). Strain A8 reduces 98.6% of 900 μg/ml Cr(VI) (Naeem et al., 2013). However, other strains like *C. cellulans* KUCr3 and CrK16 showed moderate reduction efficiencies of 40% and 41%, respectively (Chatterjee et al., 2011; Rehman and Faisal, 2015). *Microbacterium* species also demonstrate promising capabilities: *M. oleivorans* A1 reduced 750 μM Cr(VI) in 85 h (Sarkar et al., 2015), whereas *M. testaceum* B-HS2 exhibited exceptional tolerance up to 48 mM (Elahi et al., 2019). Both *Microbacterium* sp. M5 and *M. paraoxydans* SCRB19 reduced 400 and 500 ppm Cr(VI), respectively, although with different efficiencies (Kumar and Saini, 2019; Mishra et al., 2021).

Other genera also contribute to Cr(VI) bioremediation. *Brucella* sp. showed complete reduction of 50 ppm Cr(VI) using cell-free extracts (Thacker et al., 2007), and *B. intermedius* TJ-5 demonstrated bioremediation potential (Chen et al., 2022). *Exiguobacterium acetylicum* demonstrated 95.4% Cr(VI) reduction at 50 mg/L concentration (Garcia da Cunha et al., 2025). *Pseudomonas* strains, such as CPSB21, exhibited high resistance (up to 700 ppm) and reduction efficiency (up to 90%; Gupta et al., 2018; Kumar et al., 2023), with MAI4 identified as a potent reducer (Wani et al., 2019). *Kocuria* species, particularly the radio-resistant ASB107, are known for effective biosorption of Cr(VI) (Akbarpour Nesheli et al., 2017), whereas *Klebsiella pneumoniae* MS15 and *K. aerogenes* MUM reduced Cr(VI) up to 80 and 600 ppm, respectively, indicating moderate to high tolerance (Kumar et al., 2023). *Brevibacillus borstelensis* SSAU-3T demonstrated 99% Cr(VI) removal of 400 ppm Cr(VI) at 55 °C (Agrawal et al., 2025).

Chemotaxis of chromium involves the ability of microorganisms to sense and move toward or away from chromium gradients in the environment (Pandey and Jain, 2002). Some bacterial species exhibit positive chemotaxis toward Cr(VI)-contaminated regions to increase their chances of colonization and, consequently, removal of Cr(VI) (Shamim et al., 2014; Barrionuevo and Vullo, 2012). However, some microorganisms exhibit negative chemotaxis, attempting to move away from high chromium concentrations due to its toxicity (Arora et al., 2015). Chemotactic behavior is crucial for microbial bioremediation, as it guides chromium-reducing bacteria (CRB) to contaminated areas, thus enhancing detoxification efficiency. This process is regulated by transmembrane receptors, intracellular signaling pathways, and flagellar motility, which collectively increase microbial adaptation and survival under metal stress conditions (Xu et al., 2024).

Biochemical synergy between biotransformation and chemotaxis is crucial in controlling the overall efficiency of microbial Cr(VI) remediation (Pal et al., 2022). By using microbial motility and metabolic equipment, researchers are trying to create a low-cost, eco-friendly, and sustainable approach to chromium detoxification in polluted environments.

While numerous studies have explored the enzymatic and molecular mechanisms of Cr(VI) reduction in bacteria (Agrawal et al., 2025; Adhikary et al., 2025; Saxena et al., 2025), the comprehensive role of chemotaxis in influencing microbial survival and spatial behavior under Cr(VI) stress remains largely underexplored. Chemotaxis, particularly negative chemotaxis, may serve as a critical early survival mechanism by allowing bacteria to evade lethal concentrations of Cr(VI). Although *Pannonibacter phragmitetus* BB has been reported to exhibit negative chemotaxis to Cr(VI) (Chai et al., 2019), such behavioral responses have not been systematically studied across diverse taxa. In particular, *Bacillus* spp., despite being well-documented for their high Cr(VI) tolerance and reduction capabilities, lack comprehensive characterization in terms of chemotactic behavior under chromium gradients. In our previous research, we sequenced the genome of *Bacillus licheniformis* KNP (Arora et al., 2020) and confirmed its potent Cr(VI)-reducing ability (Kumar et al., 2023). However, the molecular basis and phenotypic manifestation of chemotactic response to Cr(VI) in this strain remained unexplored. The present study addresses this critical gap by linking phenotypic evidence from soft agar chemotaxis assays with genomic annotation of chemotaxis-related genes in *B. licheniformis* KNP. To the best of our knowledge, this is the first comprehensive report showing that Cr(VI)-induced negative chemotaxis in *B. licheniformis*, along with genomic evidence supporting the underlying molecular machinery. This study not only broadens the understanding of adaptive bacterial behavior under metal stress but also provides novel mechanistic insight that may help in developing more effective bioremediation strategies for chromium-contaminated environments.

## Materials and methods

### Bacterial strain

*Bacillus licheniformis* KNP was previously isolated from a sample collected from the effluent of a tannery located at Jajmau (26.4670°°N, 80.3500 °E) area of Kanpur, India (Kumar et al., 2023; Arora et al., 2020). The effluent sample was aseptically collected in a sterilized screw-capped bottle. For selective enrichment of hexavalent chromium-resistant bacteria, 1 ml of the effluent sample was inoculated into 500 ml of nutrient broth supplemented with 500 ppm potassium dichromate and incubated for 72 h at 30 °C under shaking conditions (Kumar et al., 2023; Arora et al., 2020). After 72 h of enrichment, serial dilutions of the culture were prepared up to 10^−6^ using sterile distilled water. Aliquots (100 μl) from appropriate dilutions were spread onto nutrient agar plates containing 500 ppm potassium dichromate and incubated at 30 °C for 48 h. The average microbial load was found to be ~1.3 × 10^3^ CFU/ml of enriched culture, indicating a substantial population of chromium-resistant bacteria in the tannery effluent sample. Colonies exhibiting distinct morphologies were selected; a total of 18 morphotypes were purified through repeated streaking and preserved in 10% glycerol stocks at −80 °C for subsequent analyses (Kumar et al., 2023; Arora et al., 2020). *B. licheniformis* KNP was able to transform hexavalent chromium at concentrations of 1,000 ppm K_2_Cr_2_O_7_.

### Bacterial growth in the presence of chromium

The cells of *B. licheniformis* KNP were grown in nutrient media containing various concentrations of potassium dichromate (250, 500, 750, and 1,000 ppm) under shaking conditions (160 rpm) at 30 °C. The samples were collected at regular intervals, and the optical density of the sample was determined by spectrophotometry at 600 nm.

### Chromium (VI) reduction using *B. licheniformis* strain KNP

Chromium (VI) reduction analysis was conducted in nutrient broth medium with 500 ppm K_2_Cr_2_O_7._ An overnight culture of *B. licheniformis* KNP was added to the nutrient broth medium containing 500 ppm K_2_Cr_2_O_7_. An uncultured nutrient broth enriched with 500 ppm K_2_Cr_2_O_7_ served as a control. The flasks were incubated on an orbital shaker at 160 rpm and 30 °C. At regular intervals, samples were collected, and the Cr(VI) reduction was measured using a spectrophotometric method with the 1,5-diphenylcarbazide assay (Thacker et al., 2007).

### Diphenylcarbazide (DPC) assay

To find the amount of residual Cr(VI) in the culture supernatant, we analyzed the absorbance of the purple color complex formed by Cr(VI) and 1,5-diphenylcarbazide (DPC) at 540 nm using a UV-Vis spectrophotometer. We prepared the DPC reagent by dissolving 250 mg of DPC in 50 ml of acetone. The reaction mixture included 100 or 200 μl of culture supernatant, 330 μl of 6M H_2_SO_4_, and 400 μl of DPC reagent, with the final volume brought to 10 ml using distilled water. We kept the sample at room temperature for 20 min to develop the color complex and then measured the absorbance at 540 nm (Thacker et al., 2007). To calculate the Cr(VI) reduction (%), the following formula was used:


$$\begin{array}{l} \text{\% Cr }\left(\text{VI}\right)\text{reduction }=\left(\text{Ci}-\frac{\text{Cf}}{\text{Ci}}\right)\text{x }100 \end{array}$$

[Where Ci = initial Cr(VI) concentration (mg/L) and Cf = final Cr(VI) concentration (mg/L)]

### Effects of temperature

To monitor the effects of temperature on growth and Cr(VI) reduction, strain KNP was grown on nutrient media containing 500 ppm K_2_Cr_2_O_7_ at various temperatures (20, 25, 30, and 42 °C), and samples were collected after 48 h. The growth was monitored by spectrophotometry at 600 nm, and total chromium Cr(VI) reduction was measured by the DPC assay as described above.

### Effects of pH

To monitor the effects of pH on growth and Cr(VI) reduction, strain KNP was grown on nutrient media containing 500 ppm K_2_Cr_2_O_7_ at various pH levels (5, 6, 7, 8, 9, 10, and 11), and samples were collected after 48 h. The growth was monitored by spectrophotometry at 600 nm, and total chromium Cr(VI) reduction was measured by the DPC assay as described above.

### SEM-EDX analysis

The scanning electron microscope-energy dispersive X-ray spectroscopy (SEM-EDX) analysis was performed to characterize the changes in cell morphology of bacteria in the presence of chromium. Chromium-unexposed cells were used as a control. Strain KNP was grown in nutrient broth amended with K_2_Cr_2_O_7_ as a source for Cr(VI) at a 500 ppm concentration and incubated for 48 h at 150 rpm. After incubation, the medium was centrifuged at 4 °C for 10 min at 8,000 rpm. Clear supernatant of broth was removed, and the bacterial cell pellets were washed with 0.5 M phosphate buffer saline (PBS). After washing, the cells were fixed with 2.5% glutaraldehyde for about 2 to 4 h and then washed with PBS. The cells were finally fixed with osmium tetroxide for at least 1 h. After completing these steps, cell pellets were dehydrated for 15 min with acetone at various concentrations, i.e., 30%, 50%, 70%, 90%, and 100%. Finally, the dehydrated sample was fixed or mounted with platinum by an ion sputter coater onto the carbon conductive adhesive tape before performing SEM, combined with EDX (Kumar et al., 2024).

### Fourier transform infrared spectroscopy (FTIR)

To obtain Fourier Transform Infrared spectroscopy (FTIR) spectra, strain KNP was grown overnight at 30 °C in nutrient broth amended with 500 ppm K_2_Cr_2_O_7_, while in the control, K_2_Cr_2_O_7_ was not added. After incubation, cells were centrifuged at 10,000 rpm for 10 minutes. After centrifugation, the cell pellets at the bottom were washed with 0.85% saline water, followed by double-distilled water. Washed cell pellets were dried and mixed with potassium bromide (KBr) and analyzed by an FTIR spectrophotometer (Kumar et al., 2019). The absorbance of the IR spectrum was recorded in the range of 400–4,000 cm^−1^. The spectra were recorded with 32 scans per sample at a resolution of 4 cm^−1^ to ensure adequate spectral quality for biochemical interpretation.

### Bacterial chemotaxis away from hexavalent chromium

#### Drop plate assay

The drop plate assay was performed following a method that had previously been described (Arora et al., 2015). Bacteria were cultured overnight in nutrient broth supplemented with 500 ppm potassium dichromate. The cells were centrifuged, washed twice with saline solution, and suspended in a minimal medium containing 0.3% agar. This suspension was poured onto a Petri dish and allowed to solidify. To assess bacterial attraction and repellence to chromium, a small quantity of potassium dichromate or glucose (positive control) was placed in the center of the Petri dish. The plates were incubated for a few hours. Bacterial attraction was indicated by visible rings forming around the crystals, while negative chemotaxis resulted in bacteria dispersing away from the compound.

### Chemical-in-plug method

In the chemical-in-plug method, the bacterial solution was prepared using the same protocol as for the drop plate assay (Arora et al., 2015). This solution was then poured around hard agar plugs made of minimal media, 2% Bacto agar, and either potassium dichromate (500 ppm) or glucose (100 ppm) as a positive control. The plates were incubated at 30 °C for 6 h after the plugs had solidified, and then checked for chemotactic activity.

### Identification of genes involved in chemotaxis

The draft genome of *Bacillus licheniformis* KNP was previously submitted to DDBJ/ENA/GenBank under the accession JACDXS000000000 (Arora et al., 2020). To identify the chemotactic genes, the KNP genome was annotated using the NCBI Prokaryotic Genome Annotation Pipeline (PGAP; Arora et al., 2020).

### Statistical analysis

All the experiment setup and data analysis were performed in triplicate (*n* = 3) to reduce analytical errors, and the results were represented as mean ± standard deviation (SD).

## Results and discussion

### Growth and Cr(VI) reduction studies

**Figure 1:**
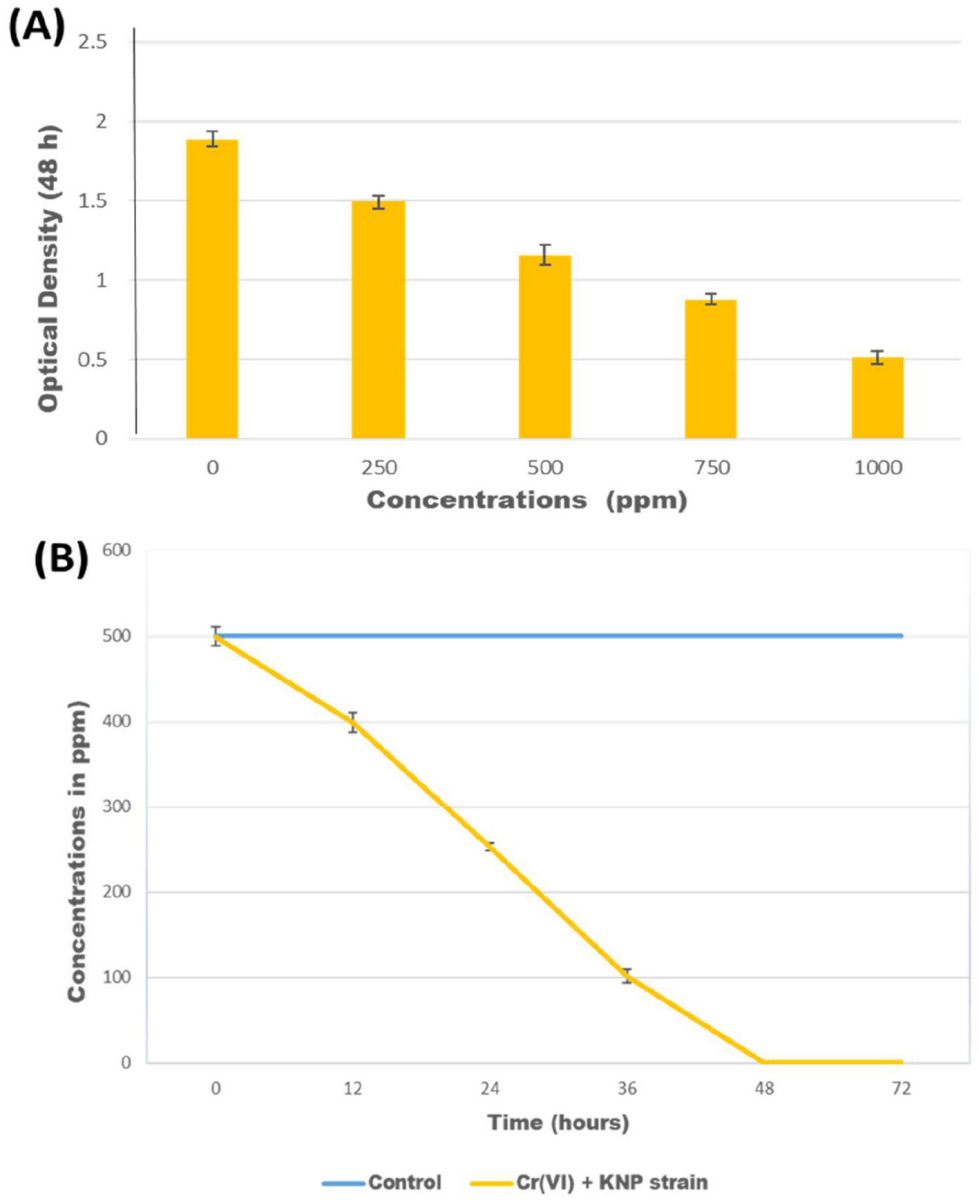
**(A)** Optical density of culture of *Bacillus licheniformis* KNP after growth for 48 h in media containing various concentrations of K_2_Cr_2_O_7_; **(B)** Reduction of Cr(VI) in concentrations of 500 ppm K_2_Cr_2_O_7_ by *Bacillus licheniformis* KNP.

The growth of *Bacillus licheniformis* strain KNP was monitored in nutrient broth containing various concentrations of K_2_Cr_2_O_7_ (250, 500, 750, and 1,000 ppm) as a source of Cr(VI). As the concentration of chromium increased, the bacterial growth was reduced. In the presence of chromium, the maximum bacterial growth was observed when the K_2_Cr_2_O_7_ concentration was 250 ppm (Figure 1A). When exposed to 1,000 ppm K_2_Cr_2_O_7_, *Bacillus licheniformis* strain KNP showed extremely reduced growth. In comparison, K_2_Cr_2_O_7_ concentrations from 250 and 500 ppm had only a mild impact on bacterial growth, whereas a concentration of 750 ppm resulted in a significant reduction in bacterial growth, indicating that higher concentrations of Cr(VI) are more toxic to bacterial growth. Previous studies also reported that bacterial growth was reduced at high concentrations of Cr(VI) (Kumar et al., 2023). Liu et al. (2006) reported that the cells of *Bacillus* sp. could not grow when exposed to 100 ppm chromium, whereas at 80 ppm, bacterial growth was reduced compared to concentrations of 5, 10, 20, and 40 ppm. Upadhyay et al. (2017) observed that *Bacillus subtilis* MNU16 grew more slowly at higher chromium concentrations (250 and 300 mg/L) compared to 50, 100, 150, and 200 mg/L. Wang et al. (2025) reported that *Microbacterium oxydans* strain S-1 exhibits robust growth (OD600 > 5.03) under Cr(VI) stress, highlighting its strong bioremediation potential.

*Bacillus licheniformis* KNP completely reduced chromium (500 ppm K_2_Cr_2_O_7_) within 48 h, whereas in the control, no reduction of chromium was observed (Figure 1B). *B. licheniformis* KNP was found to be more effective than any other *Bacillus* species reported for chromium reduction due to its ability to reduce 100% chromium at high concentrations. *Bacillus subtilis* MNU16 reduces Cr(VI) by 75% when exposed to 50 mg/L (Upadhyay et al., 2017). Zhao et al. (2012) reported a 96.85% reduction of Cr(VI) by *Bacillus cereus* when the initial Cr(VI) concentration was < 50 mg/L. Another report showed that 96.7% and 72.1% of Cr(VI) could be reduced by *Bacillus cereus* after the initial addition of 60 and 70 mg/L of Cr(VI), respectively (Murugavelh and Mohanty, 2013). After an initial exposure to 2 mM of Cr(VI), the *B. cereus* D strain reduced 87.8% of the metal in 24 h (Li et al., 2020). Wang et al. (2025) reported that *Microbacterium oxydans* strain S-1 efficiently reduced Cr(VI), achieving complete detoxification within 48–72 h depending on the initial concentration.

### Effects of temperature

**Figure 2:**
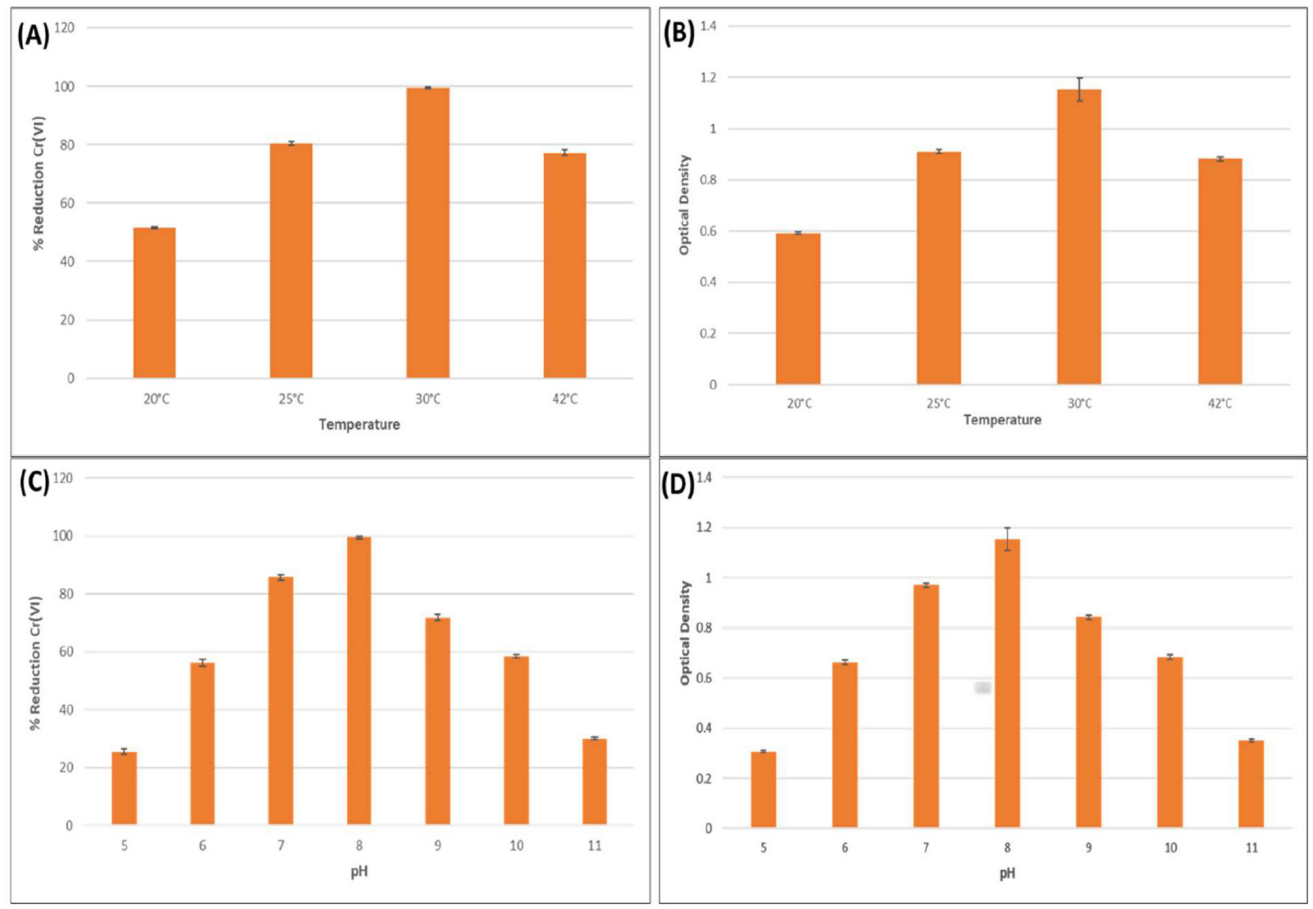
Effects of various temperatures and pH on bacterial growth and hexavalent chromium reduction. Effect of various temperatures (20, 25, 30, and 42 °C) on **(A)** hexavalent chromium reduction, **(B)** bacterial growth. Effects of various pH (5, 6, 7, 8, 9, 10, and 11) on **(C)** hexavalent chromium reduction **(D)** bacterial growth.

The effect of temperature on the Cr(VI) reduction efficiency and growth of *Bacillus licheniformis* KNP was evaluated over a 48-h incubation period. The results showed a clear temperature-dependent pattern in both chromium detoxification and bacterial proliferation. Maximum Cr(VI) reduction (99.47%) was observed at 30 °C, accompanied by the highest optical density (OD = 1.153), indicating optimal growth and metabolic activity under mesophilic conditions (Figures 2A, B). At 25 °C, Cr(VI) reduction remained substantial (80.47%) with a moderate growth level (OD = 0.911), suggesting that enzymatic activity and biomass accumulation are still effectively maintained near the optimal range. A further increase in temperature to 42 °C resulted in a decline in both reduction efficiency (77.33%) and OD (0.883), indicating the onset of thermal stress, which likely impaired chromate reductase activity and membrane integrity. The lowest reduction efficiency (51.6%) and growth (OD = 0.593) occurred at 20 °C, indicating suboptimal enzymatic kinetics and reduced cellular metabolism under cold stress. Wang et al. (2025) reported that *Microbacterium oxydans* strain S-1 efficiently reduced over 94.8% of 100 mg·L^−1^ Cr(VI) within 48 h at 30 °C. Due to the extreme temperatures, bacterial growth is adversely affected, thereby negatively affecting Cr(VI) bacterial reduction. Specifically, low temperatures inhibited cell growth more strongly by decreasing the fluidity of the cell membrane and affecting the transport system, implying that the substrate cannot enter the cell quickly to promote cell growth. Increasing temperatures may alter membrane structure and inhibit reductase and protein synthesis mechanisms (Kathiravan et al., 2010). It is therefore important that the right temperature be maintained in order to facilitate Cr(VI) reduction.

These findings align with other temperature-optimization studies in chromium bioremediation. For instance, *Staphylococcus succinus* subsp. *succinus* AMG-D1 demonstrated complete Cr(VI) removal at 200 mg/L within 120 h at 35 °C, reinforcing the importance of mesophilic conditions (Harboul et al., 2025). Similarly, *Bacillus subtilis*, when immobilized in alginate beads, showed effective Cr(VI) reduction from contaminated soil with optimal performance at pH 6 and 40 °C (Alsamhary, 2025). Although slightly higher than the optimal temperature observed for *B. licheniformis* KNP, this highlights the species-specific thermal tolerance ranges. The use of immobilization likely contributes to this enhanced thermal stability by providing physical protection to bacterial cells and supporting enzyme retention. Immobilization matrices such as alginate have been shown to buffer external temperature fluctuations, thus preserving enzymatic conformation and reducing potential damage at higher thermal conditions.

The effect of temperature on the chromium-reducing efficiency of various bacterial strains was evaluated to determine optimal conditions for Cr(VI) bioremediation. *Bacillus subtilis* MNU16 was tested for its Cr(VI) reduction capability under a range of incubation temperatures (20, 25, 30, 37, and 42 °C) over 72 h (Upadhyay et al., 2017). The results demonstrated a strong temperature dependence, with only 32.4% Cr(VI) reduction at 20 °C, which increased to 55.8% at 25 °C. A significant enhancement was observed at 30 °C, where the strain reduced nearly 75% of Cr(VI), and the maximum reduction (>90%) occurred at 37 °C, indicating this as the optimal temperature for enzymatic chromium reduction activity. However, a decline in efficiency (~68%) was recorded at 42 °C, suggesting the onset of thermal stress and possible enzyme denaturation at higher temperatures (Upadhyay et al., 2017). Similar trends were observed with other bacterial strains. *Enterococcus italicus*, immobilized in alginate beads, achieved a high reduction efficiency of 91% at 35 °C within just 2 h, which further improved to 94% with glucose supplementation, highlighting its rapid and thermally optimized detoxification potential (Srivastava et al., 2025a). Likewise, *Klebsiella* sp. (BH-A1), when immobilized in alginate beads containing 1000 mg/g biomass, showed maximum Cr(VI) reduction (87%) at 30 °C. Reduction declined slightly at 25 °C (62%) and 35 °C (78%), confirming 30 °C as the optimal range for chromate reductase activity in this strain as well (Srivastava et al., 2025b). Furthermore, *Priestia megaterium* strain BM.1 and its immobilized composite form (BM.1-Ca) were assessed over the 20 °C to 40 °C range. The BM.1-Ca composite consistently outperformed free cells, with peak reduction occurring at 35 °C. However, a decrease in activity was noted at 40 °C, possibly due to heat-induced inactivation of microbial enzymes, particularly chromate reductases. The immobilization matrix likely conferred additional thermal protection and stability, enhancing bioremediation performance across temperature ranges (Wu et al., 2025). Collectively, these results confirmed that mesophilic conditions, particularly in the 30–37 °C range, are most conducive to bacterial Cr(VI) reduction. They also underscore the role of immobilization techniques in improving thermal resilience and functional efficiency of bioremediating strains.

### Effects of pH

The influence of pH on *Bacillus licheniformis* performance was assessed to determine its Cr(VI) reduction efficiency and growth at 500 ppm K_2_Cr_2_O_7_ over 48 h. The results showed a distinct pH-dependent trend, where both bacterial growth and Cr(VI) detoxification were significantly influenced by the pH of the medium. Maximum growth (OD600 = 1.153) and Cr(VI) reduction (99.47%) were observed at pH 8, suggesting that slightly alkaline conditions favored both cell proliferation and enzymatic activity essential for chromate reduction (Figures 2C, D). This could be attributed to enhanced membrane stability, optimal enzyme conformation, and improved electron transport under these conditions. In contrast, both growth and reduction efficiencies declined sharply at pH extremes. At pH values 5 and 11, the OD600 dropped to 0.306 and 0.350, respectively, while Cr(VI) reduction fell to 25.47% and 30.07%. These findings indicate that highly acidic or alkaline conditions disrupt cellular homeostasis and likely denature chromate reductase enzymes or hinder electron donor availability, thereby impairing reduction pathways. These observations are consistent with findings from other studies. For example, *Enterococcus italicus* immobilized in alginate beads exhibited optimal Cr(VI) reduction (91%) at neutral pH 7 within 2 h, highlighting the sensitivity of reduction efficiency to pH changes (Srivastava et al., 2025a). Similarly, *Klebsiella* sp. (BH-A1) demonstrated maximum Cr(VI) reduction (87%) at pH 7 and 30 °C, with reduced performance under both acidic and alkaline conditions (Srivastava et al., 2025b). While *Bacillus wiedmannii* showed the highest Cr(VI) removal efficiency of 70.27% at pH 8, indicating that slightly alkaline conditions are optimal for chromate reduction. Similarly, bacterial growth was also enhanced at this pH, suggesting a positive correlation between cell proliferation and Cr(VI) detoxification under favorable pH conditions (Adhikary et al., 2025). *Bacillus paramycoides* S48 chromate reductase exhibited 100% activity retention at pH 7 and high enzymatic stability across the range of pH 6.0–8.0, while activity sharply declined outside this window (Kalsoom et al., 2025). Conversely, for Cr(III) removal, *Pseudomonas atacamensis* M7D1 showed the maximum adsorption at pH 4, indicating a different biosorption mechanism through extracellular polymeric substances (EPS), which is more effective under acidic conditions (Tarahomi et al., 2025). These comparative results support the conclusion that a narrow pH range, typically near-neutral to slightly alkaline, is ideal for bacterial growth and efficient Cr(VI) detoxification in bioremediation systems.

### SEM analysis and EDX analysis

**Figure 3:**
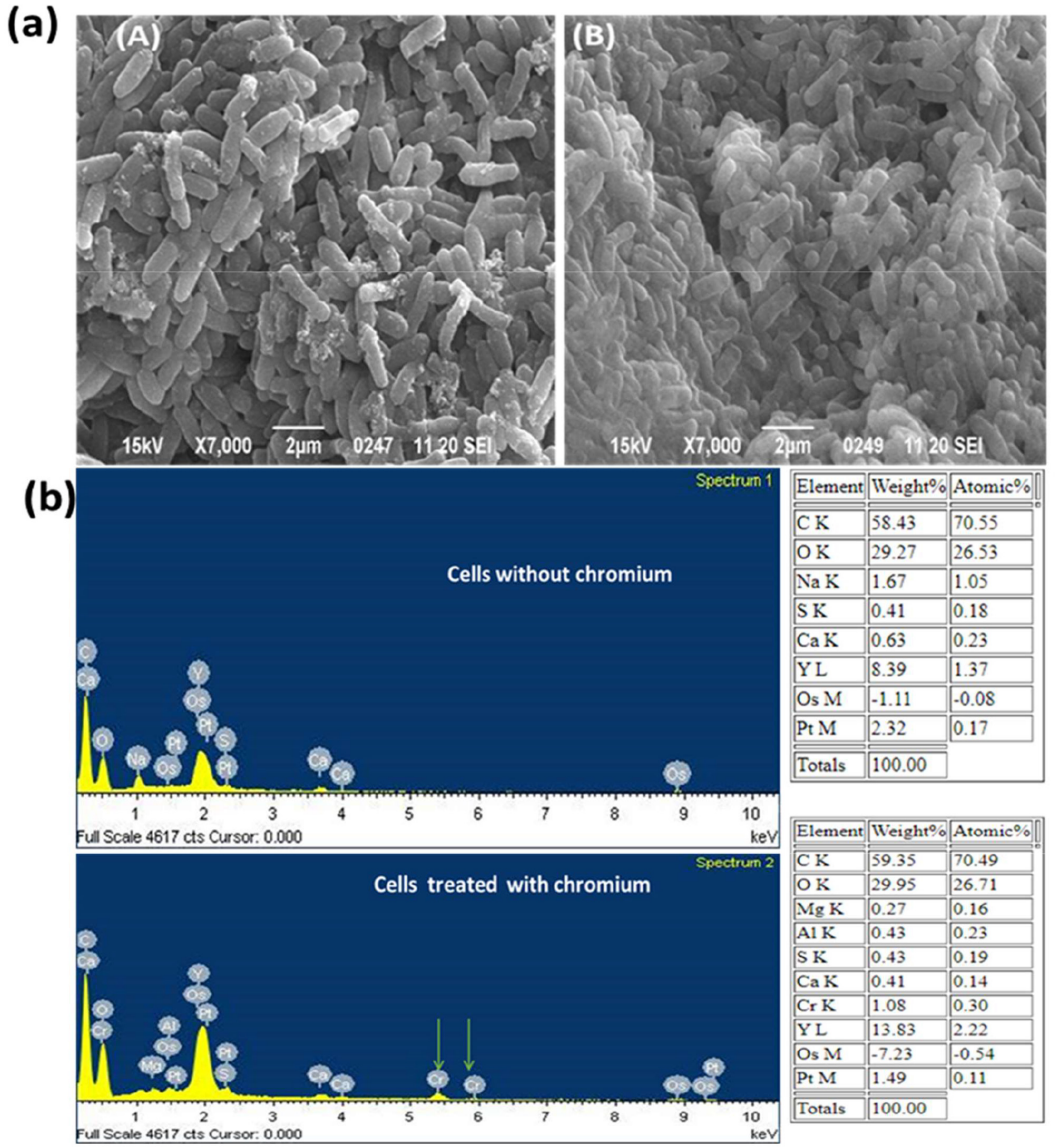
**(a)** SEM analysis of chromium untreated **(A)** and chromium treated cells **(B)** of *B. licheniformis* strain KNP; **(b)** Energy Dispersive X-ray (EDX) analysis of chromium untreated and chromium treated cells *of B. licheniformis* strain KNP.

In SEM-EDX analysis, Cr(VI) was shown to affect bacterial morphology and accumulate on cell surfaces. Cells of the strain were regular rod-shaped in the control, and there was no aggregation of cells (Figure 3aA). Furthermore, no Cr diffraction peaks were observed in the EDX spectrum. However, when stressed with Cr (VI), the cells showed imperfections, clusters, and aggregation (Figure 3aB), and the characteristic Cr peaks were found in the EDX spectrum (Figure 3b). Tan et al. (2020) observed the same result in *Bacillus* sp. CRB-B1 under Cr(VI) stress. In addition, *Escherichia* sp. TH-1 exhibited aggregation and rough surfaces after exposure to Cr(VI) (Wang et al., 2022). The aggregation and irregular cell morphology were also observed in Cr (VI)-treated cells of *Sinorhizobium* sp. SAR1 (Jobby et al., 2019). A part of bacteria, the fungus *Trichoderma lixii* CR700 also exhibited aggregation of mycelia during chromium stress (Kumar and Dwivedi, 2019). Wu et al. (2019) reported that cell deformation was caused by Cr adsorption.

The process of bacterial aggregation is considered a survival and detoxification mechanism under chromate stress. Additionally, bacteria secrete more extracellular materials under heavy metal stress (Karthik et al., 2017; Wu et al., 2019). Jobby et al. (2019) observed that *Sinorhizobium* sp. SAR1 secretes a large amount of capsular material in response to chromium exposure, resulting in increased cell size. An increase in size and the secretion of extra polysaccharide substances were also observed in a variety of bacteria under chromate stress. However, no increases in bacterial cell size were observed in this study.

Energy-dispersive X-ray spectroscopy (EDX) was used to assess the elemental profile of *Bacillus licheniformis* KNP following exposure to hexavalent chromium [Cr(VI)]. The resulting spectra indicated a chromium content of 1.08 % (by weight), suggesting limited surface binding of the metal. This low surface association implies that most of the chromium either penetrated the cells or was transformed intracellularly, rather than forming substantial extracellular precipitates.

This observation is supported by a related study by Ramli et al. (2025), who used EDX to examine *Bacillus* sp. S1 treated with chromium. Their findings showed a similarly low Cr peak (0.9% by weight) on the bacterial surface, suggesting minimal extracellular chromium accumulation. Based on these data, Ramli et al. (2025) proposed that the visible precipitate seen on the surface corresponds to untransformed Cr(VI). That chromium reduction does not occur extracellularly, implying that the bacterial cells likely reduce Cr(VI) internally.

Thus, in both cases, EDX confirmed only minimal Cr accumulation on the cell surface, supporting the conclusion that chromium reduction or detoxification processes likely occur within the bacterial cell, rather than outside it.

### FTIR analysis

**Figure 4:**
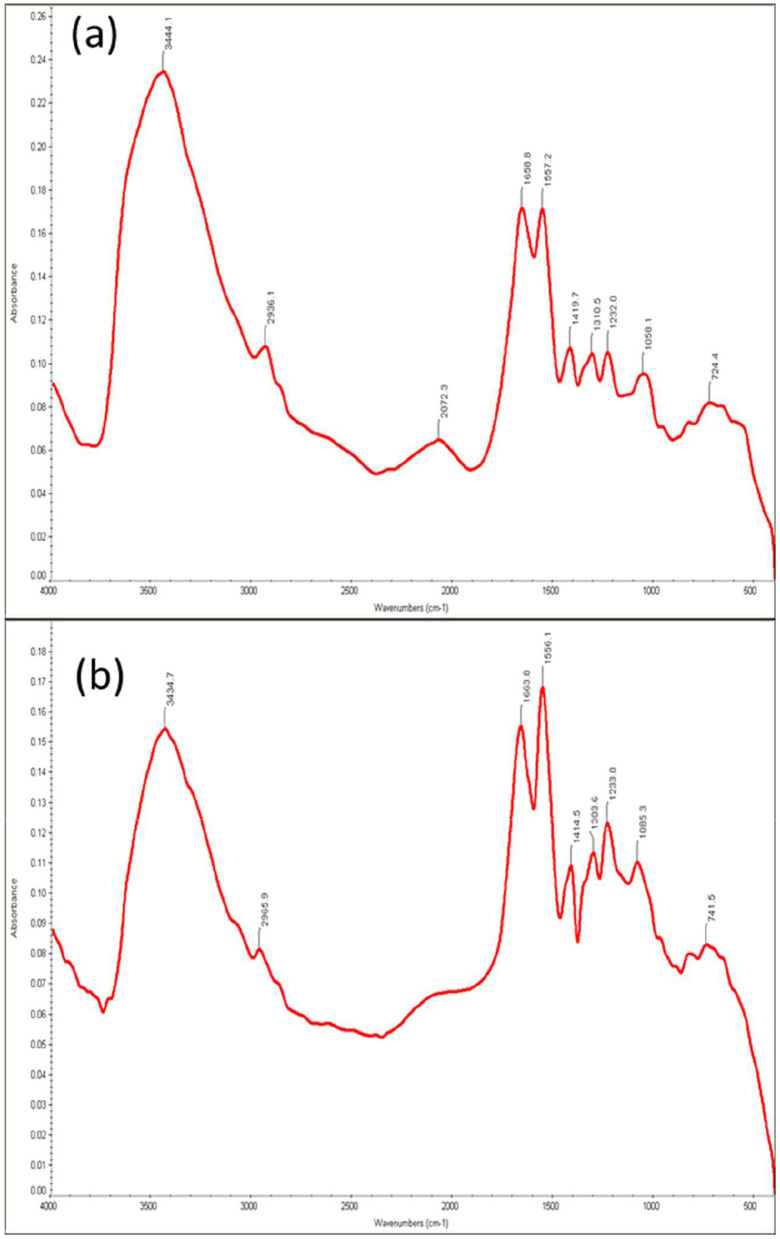
FTIR analysis of cells of *Bacillus licheniformis* KNP **(a)** without Cr(VI) treatment and **(b)** with Cr(VI) treatment.

The FTIR spectroscopy analysis was performed to identify the functional groups and chemical bonds involved in the biosorption of hexavalent chromium. The FTIR analysis of *Bacillus licheniformis* KNP with and without chromium is shown in Figure 4. Infrared spectra of *B. licheniformis* KNP in the absence of chromium stress showed characteristic absorption peaks of an amine (–NH_2_), bonded and non-bonded hydroxyl (O–H) groups, amide (–CONH–), carboxyl (–COOH), aromatic (C = C) ring, alkenes (–C = C), aliphatic (–CH_2_) groups, etc., which confirmed the presence of corresponding groups on the cell surfaces (Figure 4a). The peak at 3444.1 cm^−1^ is attributed to the presence of amine N–H stretching or the presence of a hydroxyl group (O–H) in that region. The absorption peak at 2936.1 cm^−1^ was attributed to aliphatic (–CH2) groups, which indicates C–H asymmetric stretching. The FTIR spectra peaks at 1658.8, 1557.2, 1419.7, 1310.5, 1232, and 1058.1 cm^−1^ correspond to carboxyl group (C=O), amide (–CONH–), hydroxyl (–OH) groups, –NO, aromatic amine (C–N), and phosphate group, respectively. The peak at 724 cm^−1^ can be assigned to C–H out-of-plane bending on the disubstituted benzene ring. However, when the strain KNP was subjected to chromium stress, changes in peaks and peak intensities were observed. Under chromium stress, FTIR peaks of *B. licheniformis* KNP shifted from 3444.1 to 3434.7 cm^−1^, 2936.1 to 2965.9 cm^−1^, 1658.8 to 1663.8 cm^−1^, 1557.2 to 1556.1 cm^−1^, 1419.7 to 1414.5 cm^−1^, 1310.5 to 1303.6 cm^−1^, 1232 to 1233 cm^−1^, 1058.1 to 1085.3 cm^−1^, and 724.4 to 741.5 cm^−1^. In Cr (VI)-treated bacterial cells, these variations were primarily caused by carboxyl, amino, sulfonate, and hydroxyl functional groups, which are chemically reactive with Cr ions and form permanent bonds with Cr. Table 1 summarizes the observed FTIR spectral peak shifts in *Bacillus licheniformis* KNP before and after Cr(VI) exposure, indicating interactions between functional groups on the bacterial surface and chromium ions. Adhikary et al. (2025) also reported that, under Cr(VI) stress, spectral analysis showed changes in peak absorption and intensity due to interactions between Cr(VI) and functional groups on the bacterial cell surface. However, Wang et al. (2025) observed no change in intensity of the peaks in the spectra before and after treatment in cells of *Microbacterium oxydans* strain S-1, suggesting that there was no noticeable variation in the types of functional groups identified across the samples of strain S-1.

**Table 1:** FTIR peak shifts in *Bacillus licheniformis* KNP under Cr(VI) stress.

**Peak (before)**	**Peak (after)**	**Shift direction**	**Functional group**	**Reason for shift**
3444.1 cm^−1^	3434.7 cm^−1^	Downshift	O–H/N–H stretching	Involvement of hydroxyl or amine groups in hydrogen bonding or complexation with Cr(VI)
2936.1 cm^−1^	2965.9 cm^−1^	Upshift	C–H stretching (aliphatic)	Possible conformational change or stress on the lipid bilayer due to Cr binding
1658.8 cm^−1^	1663.8 cm^−1^	Upshift	Amide I (C=O stretching)	Interaction of amide carbonyl groups with Cr(VI) ions
1557.2 cm^−1^	1556.1 cm^−1^	Slight downshift	Amide II (N–H bending)	Weak coordination between nitrogenous groups and Cr ions
1419.7 cm^−1^	1414.5 cm^−1^	Downshift	COO^−^ symmetric stretching	Carboxyl groups may be participating in metal binding
1310.5 cm^−1^	1303.6 cm^−1^	Downshift	C–N stretching (proteins)	Protein conformational changes due to metal interaction
1232 cm^−1^	1233 cm^−1^	Slight upshift	P=O or C–O stretching	Possible interaction of phosphate or ether groups
1058.1 cm^−1^	1085.3 cm^−1^	Upshift	C–O–C or PO43^−^ stretching	Stronger bonding of Cr(VI) with phosphate/carbohydrate groups
724.4 cm^−1^	741.5 cm^−1^	Upshift	C–H bending (aromatic/aliphatic)	Alteration in the hydrocarbon environment due to Cr adsorption

### Chemotaxis away from chromium

This research evaluated the chemotactic movement of *Bacillus licheniformis* strain KNP through the drop plate assay and chemical plug technique to assess its reactions to Cr(VI) and glucose. The two techniques presented information regarding the direction of movement of the strain toward or away from chemical stimuli.

### Negative chemotaxis toward Cr(VI)

**Figure 5:**
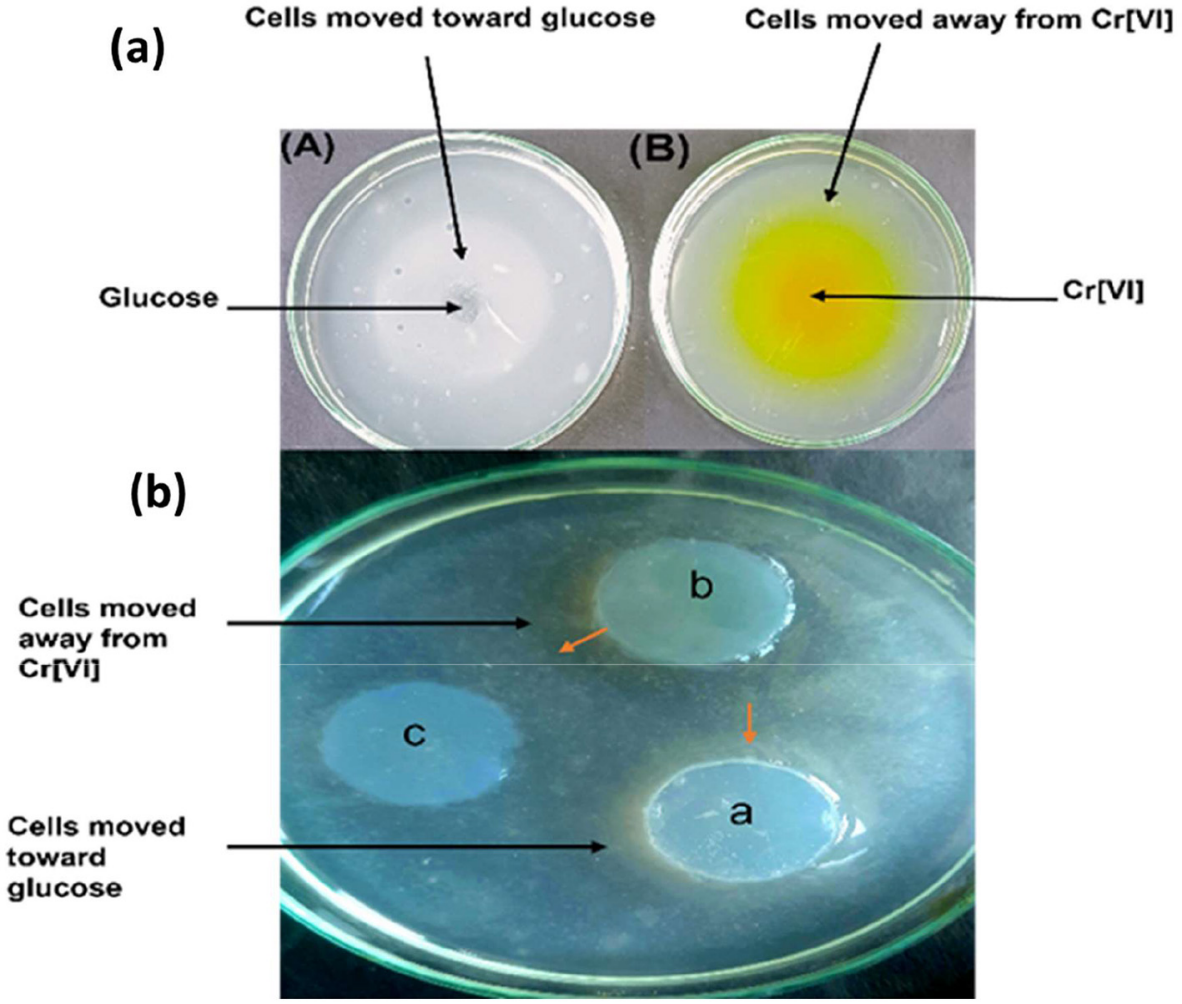
Chemotaxis away from chromium. **(a)** Drop plate assay showing accumulation of KNP cells toward glucose **(A)** and movement of KNP cells away from hexavalent chromium **(B)**. **(b)** Chemical in Plug assay showing accumulation of KNP cells toward glucose (a). Movement of KNP cells away from hexavalent chromium (b), where no chemotactic movement is observed toward agar plug without chromium or glucose.

In the drop plate assay, *Bacillus licheniformis* strain KNP exhibited a clear avoidance response to Cr(VI), forming a well-defined zone of separation around the chromium crystal (Figure 5aB). This indicates that Cr(VI) acts as a repellent, triggering a negative chemotactic response in the strain. The same was observed under the chemical plug assay, where KNP cells migrated away from the Cr(VI) agar plug actively and formed a comparable clear zone (Figure 5bb). These results are consistent with previous studies that demonstrated that microorganisms react to several toxic compounds, such as heavy metals, hydrocarbons, and oxidizing agents like hydrogen peroxide, hypochlorite, and N-chlorotaurine, with negative chemotaxis (Tso and Adler, 1974; Young and Mitchell, 1973; Ohga et al., 1993; Benov and Fridovich, 1996). This avoidance response is considered a crucial survival strategy, where microbes avoid environments with poisonous levels of chemicals. The negative chemotaxis of *B. licheniformis* strain KNP toward Cr(VI) suggests its potential application in bioremediation. Although this avoidance behavior can initially be repulsive to microbial colonization of polluted sites, the ability of the strain to metabolize or immobilize chromium remains exploitable to reduce environmental pollution. A study of the metabolic pathways and control mechanisms governing this chemotactic response may further optimize the strategic use of *B. licheniformis* in bioremediation.

### Positive chemotaxis toward glucose

In contrast to its response to Cr(VI), strain KNP showed positive chemotaxis to glucose in both procedures. For the drop plate assay, cells migrated toward the source of glucose with a dense accumulation around it (Figure 5aA). The same was observed for the chemical plug assay, where the strain migrated toward the glucose agar plug to create an accumulation ring (Figure 5ba). This response highlights glucose as a strong attractant, likely due to its role as an energy source used in promoting bacterial growth and metabolism. Positive chemotaxis to glucose emphasizes the ability of the strain to detect and migrate toward nutrient-rich environments, which is essential for survival and colonization, particularly under conditions of nutrient limitation. This characteristic is beneficial in bioremediation strategies because organic nutrients can be supplemented to induce microbial growth and maximize detoxification efficiency. The glucose chemotactic response may also facilitate co-consumption of Cr(VI) and organic substrates, maximizing bioremediation efficiency in mixed-contaminant systems. These findings confirmed that *Bacillus licheniformis* strain KNP has special chemotactic activities: negative chemotaxis from Cr(VI), and positive chemotaxis toward glucose. These characteristics suggest that the strain KNP can be utilized for bioremediation, as its ability to repel toxic heavy metals while migrating toward nutrient-rich zones can be utilized to enhance remediation procedures. Further studies should focus on clarifying the molecular and genetic foundations of these chemotactic processes to further develop the environmental potential of the strain.

### Annotation of chemotactic genes

The identification of chemotaxis-specific genes in *Bacillus licheniformis* strain KNP provides us with insight into the mechanisms of its chemotactic responses to glucose and Cr(VI) at the molecular level. The genes encode proteins that participate in sensing chemical gradients, transduction, and regulation of motility. Bacterial chemotaxis is a highly regulated process by particular proteins like methyl-accepting chemotaxis proteins (MCPs) and chemotaxis (Che) proteins that facilitate signal perception, transduction, and movement based on environmental signals (Bi and Sourjik, 2018). MCPs are transmembrane receptors and are the primary sensors for chemical gradients. MCPs in *Escherichia coli* detect attractants or repellents and convey signals to downstream components of the chemotaxis signaling pathway (Rollins and Dahlquist, 1981; Parkinson et al., 2015). Specific MCPs have been identified in various bacteria that respond to particular compounds, such as NtdY and NbaY, which function as MCPs for 2-nitrotoluene and 2-nitrobenzoate in *Acidovorax* strain JS42 (Rabinovitch-Deere and Parales, 2012) and *Pseudomonas fluorescens* strain KU-7, respectively (Iwaki et al., 2007). Table 2 summarizes a list of annotated chemotactic genes in *Bacillus licheniformis* KNP.

**Table 2:** A list of annotated chemotactic genes in the genome of *Bacillus licheniformis* KNP.

**Gene**	**Protein**	**Role in chromium chemotaxis**
MBA1159711.12 MBA1159841.1 MBA1159906.1 MBA1160874.1 MBA1161432.1 MBA1162509.1 MBA1162510.1 MBA1162511.1 MBA1163489.1	Methyl-accepting chemotaxis proteins (MCPs)	Detects chromium ions as a repellent
MBA1161127.1	CheA	Autophosphorylates and transfers phosphate to CheY to mediate motor response
MBA1161128.1	CheW	Couples MCPs to CheA for efficient signal relay
MBA1160884.1	CheV	Assists in signal coupling and fine-tunes CheA activation
MBA1161129.1	CheC	Regulates adaptation by interacting with response regulators like CheY
MBA1161130.1	CheD	Demethylates MCPs, modulating their sensitivity to chromium
MBA1161126.1	Response regulator (CheB-like)	Removes methyl groups from MCPs for adaptation to persistent chromium signals

**Figure 6:**
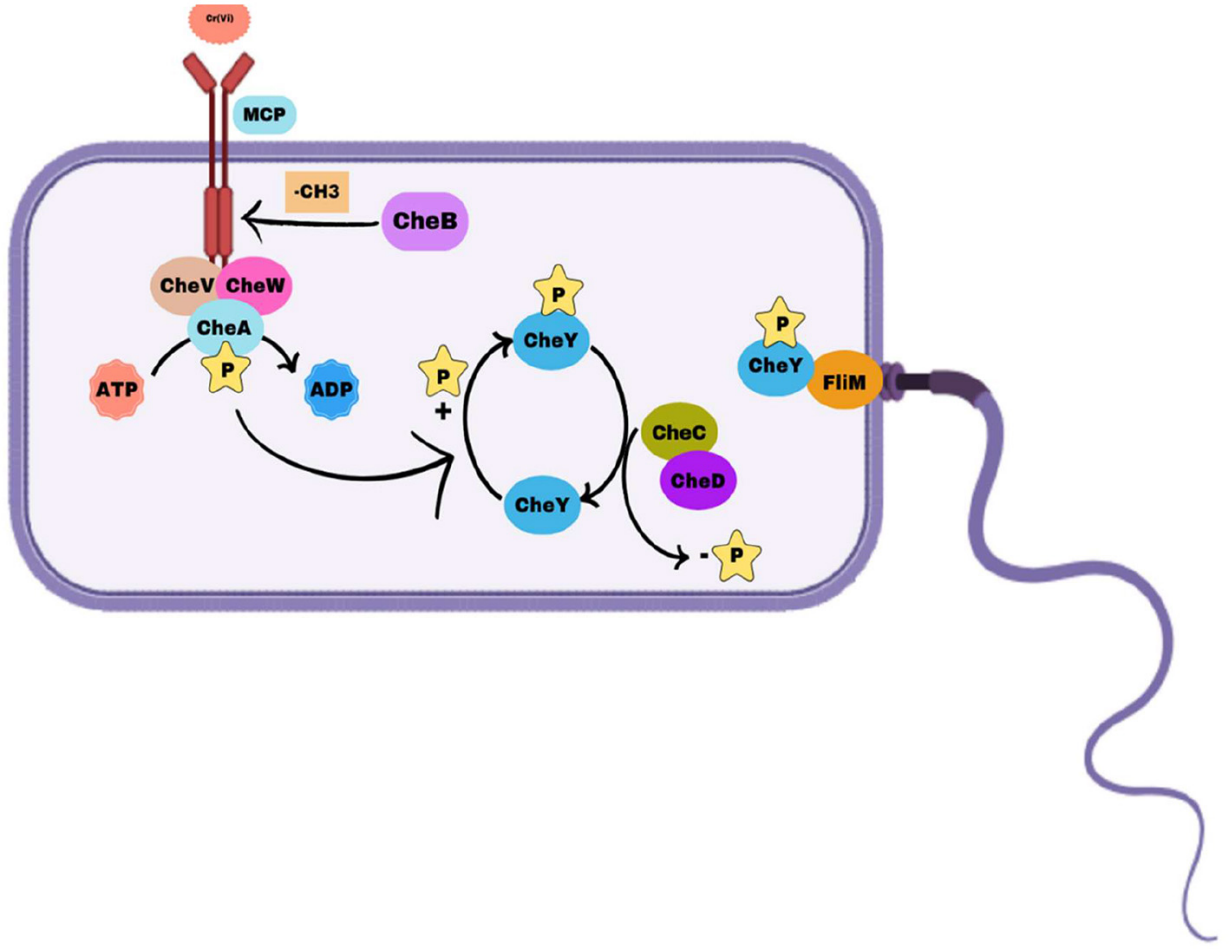
Mechanism of chemotaxis in *Bacillus licheniformis* strain KNP. Methyl-accepting chemotaxis proteins (MCPs) detect environmental signals and interact with CheW and the histidine kinase CheA to form a signaling complex. Upon activation, CheA autophosphorylates and transfers the phosphoryl group to the response regulator CheY. Phosphorylated CheY (CheY-P) binds to the flagellar motor protein FliM, inducing clockwise (CW) rotation and tumbling, whereas dephosphorylated CheY allows counterclockwise (CCW) rotation for smooth swimming. CheC and CheD modulate the dephosphorylation of CheY-P, influencing motor control, whereas CheV plays a role in signal adaptation by linking MCP signaling with the phosphorylation system. Methyl esterase dynamically regulates MCP activity, ensuring efficient bacterial navigation in response to environmental changes.

Based on identified chemotactic genes, we proposed a mechanism of negative chemotaxis of chromium in the *Bacillus licheniformis* strain KNP (Figure 6).

#### Sensing chromium as a repellent

Methyl-Accepting Chemotaxis Proteins (MCPs) like MBA1159711.1, MBA1159841.1, MBA1159906.1, and others detect chromium ions [e.g., Cr(VI)] as a repellent through their extracellular ligand-binding domains. The binding of chromium induces a conformational change in the MCPs, activating their intracellular signaling domains.

#### Signal relay and activation of CheA

The activated MCPs interact with CheW (MBA1161128.1) to form a complex that connects MCPs to the histidine kinase CheA (MBA1161127.1). The conformational change in MCPs stimulates the autophosphorylation of CheA on a conserved histidine residue. CheV (MBA1160884.1) assists in coupling the MCP activity to CheA, fine-tuning the response.

#### Phosphotransfer and flagellar control

Phosphorylated CheA transfers its phosphate group to the response regulator protein CheY (not listed but assumed present in strain KNP). Phosphorylated CheY interacts with the flagellar motor, inducing clockwise rotation. Clockwise rotation causes the strain to tumble, disrupting its forward motion and reorienting the cell away from the chromium source.

#### Adaptation mechanisms for persistent chromium signals

The methylation status of MCPs is dynamically regulated to enable adaptation: CheB (MBA1161126.1) removes methyl groups from MCPs, reducing their sensitivity to chromium over time. CheD (MBA1161130.1) also participates in MCP demethylation, supporting signal adaptation. CheC (MBA1161129.1) acts as a regulator, ensuring proper adaptation and preventing overstimulation of the pathway.

#### Resetting the chemotaxis machinery

As the bacterium moves away from the chromium gradient, MCPs return to their baseline state. CheB and CheD restore receptor sensitivity by demethylating MCPs, preparing the cell for future responses to chromium or other stimuli.

#### Movement away from chromium

The continuous phosphorylation and dephosphorylation cycle of CheA and CheY leads to controlled reorientation and swimming in new directions. The strain eventually develops a net movement away from the chromium source, avoiding toxicity.

## Conclusion

*Bacillus licheniformis* KNP grew well in the presence of the hexavalent chromium and completely reduced Cr(VI) within 48 h when supplemented with 500 ppm K_2_Cr_2_O_7_. Compared to the control, Cr (VI)-treated KNP cells showed a decrease in cell size, cell aggregation, and slight deformity in cell shape. Furthermore, the Cr treated cells of strain KNP accumulated a small amount of Cr on their surface. A Cr(VI) molecule interacts with functional groups and chemical bonds in KNP cells.

The strain KNP uses MCPs to detect chromium ions as a repellent. The signal is transmitted through CheW and CheA to CheY, which alters flagellar rotation and promotes reorientation. Adaptation proteins such as CheB and CheD modulate receptor sensitivity, ensuring the strain maintains effective negative chemotaxis by moving away from chromium-contaminated environments to avoid its toxic effects.

## Data Availability

The genome sequence of the bacterial strain KNP is publicly available. This data can be found here: https://www.ncbi.nlm.nih.gov/nuccore/JACDXS000000000.1/. All other data are available on request to corresponding authors.
